# Supplementary material for: Urine lipoarabinomannan concentrations among HIV-negative adults with pulmonary or extrapulmonary tuberculosis disease in Vietnam
Source: PLOS Glob Public Health. 2024 Nov 6;4(11):e0003891. doi: 10.1371/journal.pgph.0003891 (PMC11540228; doi:10.1371/journal.pgph.0003891)
Supplement: S1 Table — The presumptive PTB and presumptive EPTB groups compared to the MRS, Xpert, MGIT and the CRS were used for the diagnosis of active TB. Abbreviations: N, number; TP, true positive; TN, true negative; FN, false negative; FP, false positive; CI, confidence interval; PPV, positive predictive value; NPV, negative predictive value; PLR, positive likelihood ratio; NLR, negative likelihood ratio S/A, S4-20/A194-01; F/A, FIND28/A194-01; -PTB, pulmonary tuberculosis; MGIT, mycobacterial growth indicator tube; MRS, microbiological reference standard; CRS, clinical reference standard; EPTB, extra pulmonary tuberculosis; N/A, not applicable. $Participants with inadequate specimen, NTM, or not ordered set to missing for culture only reference standard. £1 participant excluded due to invalid result using visual assessment criteria. * 22 participants had a non-sputum sample taken for Xpert Ultra testing; participants with a sputum sample taken were excluded from analysis. The 22 shown due to having a non-sputum sample for Xpert Ultra testing are likely not a random sample of the total 53 participants in the table, and diagnostic performance estimates should be interpreted with caution. (DOCX) [file pgph.0003891.s001.docx]

|  |  | **Positive by reference standard** | | **Negative by reference standard** | |  |  |  |  |
| --- | --- | --- | --- | --- | --- | --- | --- | --- | --- |
| **Reference Standard** | **n** | **TP/TP+FN** | **Sensitivity**  **(95% CI)** | **TN/TN+FP** | **Specificity**  **(95% CI)** | **PPV**  **(95% CI)** | **NPV**  **(95% CI)** | **PLR**  **(95% CI)** | **NLR**  **(95% CI)** |
| **Presumptive PTB** |  |  |  |  |  |  |  |  |  |
| MRS | 692 | 184/335 | 0.55  (0.49, 0.60) | 277/357 | 0.78  (0.73, 0.82) | 0.70  (0.64, 0.75) | 0.65  (0.60, 0.69) | 2.45  (1.97, 3.04) | 0.58  (0.51, 0.66) |
| Xpert Ultra | 692 | 169/300 | 0.56  (0.51, 0.62) | 297/392 | 0.76  (0.71, 0.80) | 0.64  (0.58, 0.70) | 0.69  (0.65, 0.74) | 2.32  (1.90, 2.84) | 0.58  (0.50, 0.66) |
| MGIT^$^ | 655 | 159/280 | 0.57  (0.51, 0.63) | 283/375 | 0.75  (0.71, 0.80) | 0.63 (0.57, 0.69) | 0.70  (0.65, 0.74) | 2.31  (1.89, 2.84) | 0.57  (0.49, 0.66) |
| CRS | 692 | 203/414 | 0.49 (0.44, 0.54) | 217/278 | 0.78 (0.73, 0.83) | 0.77 (0.71, 0.82) | 0.51 (0.46, 0.56) | 2.23 (1.75, 2.85) | 0.65 (0.58, 0.73) |
| **Presumptive EPTB** |  |  |  |  |  |  |  |  |  |
| MRS | 53 | 3/6 | 0.50  (0.12, 0.88) | 29/47 | 0.62  (0.46, 0.75) | 0.14  (0.03, 0.36) | 0.91  (0.75, 0.98) | 1.31  (0.54, 3.14) | 0.81  (0.35, 1.86) |
| Xpert Ultra* | 22 | N/A | N/A | N/A | N/A | N/A | N/A | N/A | N/A |
| MGIT | 53 | 2/3 | 0.67  (0.09, 0.99) | 31/50 | 0.62  (0.47, 0.75) | 0.10  (0.01, 0.30) | 0.97  (0.84, 1.00) | 1.75  (0.73, 4.21) | 0.54  (0.11, 2.70) |
| CRS | 53 | 16/33 | 0.48  (0.31, 0.66) | 15/20 | 0.75  (0.51, 0.91) | 0.76  (0.53, 0.92) | 0.47  (0.29, 0.65) | 1.94  (0.84, 4.48) | 0.69  (0.45, 1.04) |
