# Supplementary material for: Urine lipoarabinomannan concentrations among HIV-negative adults with pulmonary or extrapulmonary tuberculosis disease in Vietnam
Source: PLOS Glob Public Health. 2024 Nov 6;4(11):e0003891. doi: 10.1371/journal.pgph.0003891 (PMC11540228; doi:10.1371/journal.pgph.0003891)
Supplement: S2 Table — The LLODs from the immunoassay plates used for uLAM quantitation using the S4-20/A194-01 immunoassay stratified by MRS status from the presumptive PTB group (n = 692). Abbreviations: N, number; MRS, microbiological reference standard; N/A, not applicable. (DOCX) [file pgph.0003891.s002.docx]

| **LLOD (pg/mL)** | **n (%)** | **uLAM Detected (n=139)** | | **uLAM Not Detected (n=553)** | |
| --- | --- | --- | --- | --- | --- |
|  |  | **Mean (min, max)** | **n** | **Mean (min, max)** | **n** |
| 10 | 16 | 129.5 (22, 346) | 4 | 0.1 (0,1) | 12 |
| 11 | 111 | 162.0 (12, 2160) | 27 | 1.1 (0, 8) | 84 |
| 12 | 39 | 902.8 (17, 7039) | 10 | 1.1 (0, 10) | 29 |
| 13 | 79 | 224.0 (31, 599) | 8 | 1.5 (0, 8) | 71 |
| 15 | 57 | 216.9 (15, 1691) | 13 | 1.2 (0, 10) | 44 |
| 16 | 34 | 197.2 (17, 2138) | 15 | 3.3 (0, 12) | 19 |
| 17 | 39 | 1582.7 (21, 9244) | 6 | 1.6 (0, 11) | 33 |
| 18 | 117 | 404.0 (18, 6017) | 24 | 2.1 (0, 16) | 93 |
| 19 | 37 | 12443.3 (41, 36994) | 3 | 2.4 (0, 14) | 34 |
| 21 | 75 | 82.2 (21, 299) | 16 | 1.8 (0, 16) | 59 |
| 22 | 8 | 32.0 (32, 32) | 1 | 1.9 (0, 11) | 7 |
| 23 | 38 | 653.5 (71, 1236) | 2 | 2.5 (0, 14) | 36 |
| 26 | 39 | 369.6 (27, 1235) | 7 | 3.3 (0, 18) | 32 |
| 52 | 1 | 69.0 (69, 69) | 1 | NA | 0 |
| 63 | 2 | 94.5 (68, 121) | 2 | NA | 0 |
