# Supplementary material for: Urine lipoarabinomannan concentrations among HIV-negative adults with pulmonary or extrapulmonary tuberculosis disease in Vietnam
Source: PLOS Glob Public Health. 2024 Nov 6;4(11):e0003891. doi: 10.1371/journal.pgph.0003891 (PMC11540228; doi:10.1371/journal.pgph.0003891)
Supplement: S3 Table — The LLOD from the immunoassay plates used for uLAM quantitation using the S4-20/A194-01 immunoassay stratified by MRS status from the presumptive EPTB group(N = 53). Abbreviations: N, number; MRS, microbiological reference standard; N/A, not applicable. (DOCX) [file pgph.0003891.s003.docx]

| **LLOD (pg/mL)** | **n (%)** | **LAM Detected (n=8)** | | | | **LAM Not Detected (n=45)** | | | |
| --- | --- | --- | --- | --- | --- | --- | --- | --- | --- |
|  |  | **MRS Positive**  **(n=2)** | | **MRS Negative**  **(n=6)** | | **MRS Positive**  **(n=2)** | | **MRS Negative**  **(n=6)** | |
|  |  | **Mean**  **(min, max)** | n | **Mean**  **(min, max)** | n | **Mean**  **(min, max)** | n | **Mean**  **(min, max)** | n |
| 10 | 4 (7.5) | - | 0 | - | 0 | - | 0 | 0.5 (0, 2) | 4 |
| 13 | 1 (1.9) | - | 0 | - | 0 | 10.0 (10, 10) | 1 | - | 0 |
| 16 | 4 (7.5) | 51.0 (51, 51) | 1 | 24.0 (18, 34) | 3 | - | 0 | - | 0 |
| 22 | 26 (49.1) | 50.0 (50, 50) | 1 | - | 0 | 4.0 (4, 4) | 1 | 1.9 (0, 15) | 24 |
| 27 | 18 (34) | - | 0 | 38.3 (32, 45) | 3 | 7.5 (0, 15) | 2 | 5.5 (0, 21) | 13 |
