# Supplementary material for: Urine lipoarabinomannan concentrations among HIV-negative adults with pulmonary or extrapulmonary tuberculosis disease in Vietnam
Source: PLOS Glob Public Health. 2024 Nov 6;4(11):e0003891. doi: 10.1371/journal.pgph.0003891 (PMC11540228; doi:10.1371/journal.pgph.0003891)
Supplement: S1 Text — (DOCX) [file pgph.0003891.s005.docx]

Participant inclusion and exclusion criteria

Inclusion criteria:

1. Presumed of having active pulmonary or extrapulmonary clinical TB disease by clinical team. For example, clinical symptoms of pulmonary TB (PTB): Persistent cough of two weeks or longer, unexplained fever/chills, night sweats, and weight loss. Clinical symptoms of extra-pulmonary TB (EPTB): unexplained fever/chills, night sweats, and weight loss with symptoms of the organ lesioned.
2. Being ≥18 years old.
3. Attending outpatient clinic or admitted to inpatient medical ward <72 hours.
4. Have NOT received TB preventative therapy (TPT) within prior 3 months.
5. Have NOT received anti-TB treatment for more than 24 hours (i.e. have just started treatment for TB).
6. Willing/able to provide written informed consent

Exclusion criteria:

1. Being< 18 years of age.
2. Received TPT within prior 3 months.
3. Patients who are already with diagnosed TB.
4. Received anti-TB treatment for more than 24 hours.
5. Not willing/able to provide written informed consent.
6. Patients recently diagnosed with chronic kidney dysfunction, meningitis, mental disorder, or urinary tract infections are to be excluded.
