# Supplementary material for: Urine lipoarabinomannan concentrations among HIV-negative adults with pulmonary or extrapulmonary tuberculosis disease in Vietnam
Source: PLOS Glob Public Health. 2024 Nov 6;4(11):e0003891. doi: 10.1371/journal.pgph.0003891 (PMC11540228; doi:10.1371/journal.pgph.0003891)
Supplement: S2 Text — (DOCX) [file pgph.0003891.s006.docx]

Study Procedures

Research study nurses (RSNs) identified potential participants with presumptive clinical TB disease by convenience sampling among inpatients and outpatients at the study hospital. RSNs collected patient demographic and health information, employment status, prior HIV and TB treatment, medical conditions, and current symptoms reported.

All presumptive TB patients received diagnostic testing by AFB smear, GeneXpert and/or culture as dictated by the national testing algorithms for routine care symptoms. For study specific purposes, each participant provided 1 urine specimen (50 - 100 mLs) on the day of enrollment, prioritizing an early morning, first-void urine specimen if possible. Additional urinalysis was performed. All persons who had not provided a sputum specimen (or non-respiratory sample for EPTB patients) for Xpert Ultra and TB culture testing were requested to provide sputum specimens for microbiological TB investigation. If the participant did not know their HIV status, they were offered HIV rapid testing using fingerstick. Participants identified as HIV positive were offered CD4 testing if they were newly diagnosed or had not received a CD4 count in the past 3 months. If other blood test data was available for the participant’s medical chart (glucose, HbA1c, etc.), information was collected.

Follow-up phone calls for participants were conducted at 2 and 6 months after enrollment visit to evaluate clinical status, symptoms, and assess for response to TB treatment. Clinical charts and laboratory records were reviewed to ascertain any incident diagnoses. VITIMES TB registration records was searched at 6 months to determine whether the participant was initiated on TB treatment.
