# Supplementary material for: Urine lipoarabinomannan concentrations among HIV-negative adults with pulmonary or extrapulmonary tuberculosis disease in Vietnam
Source: PLOS Glob Public Health. 2024 Nov 6;4(11):e0003891. doi: 10.1371/journal.pgph.0003891 (PMC11540228; doi:10.1371/journal.pgph.0003891)
Supplement: S1 Checklist — (DOCX) [file pgph.0003891.s007.docx]

Inclusivity in global research

PLOS’ policy on inclusivity in global research aims to improve transparency in the reporting of research performed outside of researchers’ own country or community and ensures that PLOS publications reporting global research adhere to high standards for research ethics and authorship. Authors of relevant research articles may be asked to complete the questionnaire below, which outlines ethical, cultural, and scientific considerations specific to inclusivity in global research. This questionnaire may be requested when researchers have travelled to a different country to conduct research, if research uses samples collected in another country, research with Indigenous populations or their lands, or if research is on cultural artefacts. Researchers travelling to another country solely to use laboratory equipment will not normally be required to complete the questionnaire. However, the questionnaire can be requested at the journal’s discretion for any submission – if you have been requested to complete this questionnaire by the PLOS journal you submitted to, please do so.

Please complete the questionnaire below and include this as a Supporting Information file with your manuscript. Note that if your paper is accepted for publication, this checklist will be published with your article in the supporting information files. Please ensure that you reference the checklist in the main body of your manuscript. We suggest adding a subsection ‘Inclusivity in global research’ to your Methods section and adding the following sentence: “Additional information regarding the ethical, cultural, and scientific considerations specific to inclusivity in global research is included in the Supporting Information (SX Checklist)”

The questions have been designed to be applicable to a wide range of study types, and there are subsections for both human subjects research and non-human subjects research. If any of the questions are not relevant to your research please mark them as “N/A” as appropriate.

**Ethical considerations, permits and authorship**

*This section is applicable to all research types.*

Provide details as to who granted permissions and/or consent for the study to take place in the Methods section of your manuscript. This should include the names of **all** ethics boards, governmental organizations, community leaders or other bodies that provided approval for the study. If individuals provided approval refer to these people by their role or title but do not list their name(s).

Reported on page number: The relevant text is included in the clean document, page 5, lines 94 -99. Three ethics boards were approached with the institutional review boards (IRBs) at the National Lung Hospital (NLH, Hanoi, Vietnam; reference No: 26/21/CN-HDDD) and the Vietnam Ministry of Health (Hanoi, Vietnam; reference No: 95/CN-HDDD). The PATH ethics board made their approval decision contingent on the approval by the NLH IRB.

If there were any deviations from the study protocol after approval was obtained please provide details of these changes in the Methods section of your manuscript.

Reported on page number: No deviations were made or observed during the course of the study.

Reported on page number: No deviations were made or observed during the course of the study.

Did this study involve local collaborators that are residents of the country where the research was conducted or members of the community studied? If you do not have any authors from said communities, please provide an explanation for this below.

Yes, we included 13 local collaborators from the study site (9) and operational and administrative staff of the PATH country program office (4) who helped support the study.

Everyone listed as an author should meet PLOS’ criteria for authorship and all individuals who meet these criteria should be included in the author byline, rather than the acknowledgements. For further information please see the journal’s Authorship Policy.

All listed authors meet the criteria. We did not defer any individuals to the acknowledgements.

**Human subjects research (e.g. health research, medical research, cross-cultural psychology)**

Did you obtain written informed consent from a representative of the local community or region before the research took place? How did you establish who speaks for the community? Details of written informed consent obtained from study participants should be reported separately in the Methods section of your manuscript.

Our study did not involve community involvement and so informed consent from the relevant representatives was not sought. However, each participant was only enrolled into the study once they had read and signed the consent form and were eligible for inclusion based on our criteria. For the consent process please see page 5, lines 98 – 99 of the manuscript.

How did members of the local community provide input on the aims of the research investigation, its methodology, and its anticipated outcome(s)?

We worked together with the NLH clinical staff in the drafting of the study protocol in order to gain their insights with patient flow into their facility and what was the process and protocols that worked best for them, the hospital staff and the patients. Similarly, with the test methods we relied on their inputs as to how they wanted to lead this work in their hospital. The Hanoi-based PATH staff worked to liaise with our clinical partners on preparing the study protocol and also to provide financial compensation and other necessary resources as needed by our local clinical partner. All members helped to prepare or review the manuscript.

When engaging with the local community, how did you ensure that the informed consent documents and other materials could be understood by local stakeholders?

We developed an shared Informed Consent Form protocol that was included in the submission package for IRB approval, the protocol was prepared in English and also translated into Vietnamese by a professional translator not associated with the study. It was also retranslated into English to ensure that it was fully correct. The consent form was shared with patients who were approached to enter the study. The document was made available in Vietnamese and a study registered nurse (SRN) was with each individual to answer questions and to be sure they understood the content of the form. The SRN also completed an Informed Consent Form Checklist to ensure a consistent process for enrollment. If a patient was illiterate then the SRN followed a specific protocol and read the consent form to both the prospective participant and also a witness not associated with the study. If the patient agreed to consent then they make their mark on the consent form then the witness also signed to complete the enrollment process.

Will the findings of the research be made available in an understandable format to stakeholders in the community where the study was conducted (e.g. via a presentation, summary report, copies of publications, etc.)? Please provide details of how this will be achieved.

Our goal is that this research will be made available to the stakeholders in the community as an open access online publication. PATH have placed no data access restrictions should the NLH or other partners want to use the data in our study for their own purposes. We did try to present our collective work in a poster abstract for the Union of the Lung meeting but this was declined.

**Non-human subjects research using specimens/ animals collected as part of the study, or those housed in archival collections. Examples include archaeology, paleontology, botany and zoology.**

Did the permission you obtained from a local authority to perform the study include an agreement on access to outputs and benefit sharing? This may include procedures to enable fair distribution of the benefits and resources arising from the research performed. Please include any details of Prior Informed Consent and Benefit Sharing Agreements obtained. These may be required by field-specific regulations, for example the Convention on Biological Diversity (CBD) and the associated Nagoya Protocol.

Not applicable to this study.

If the material used in your study was imported, please A) provide the year it was imported and B) indicate whether permits were obtained to import/export the materials used, C) provide details of any permits obtained. If this information is not available, please indicate this.

Urine samples were shipped in two batches from the NLH in Hanoi to the PATH laboratory in Seattle; on 04/26/22 and 07/05/2022. The export permit (No: L/KDYTQT) was approved by the Hanoi Department of Health (03/21/2022). PATH also received an import permit from the US CDC using the following import PHS Permit No. 202201018 -0181A, Issue date 01/20/2022, Expiration Date 01/20/2023.

If you used archival specimens, please state how the material used in your study was acquired by the institute it is held in and provide details of any permits obtained for the original excavations/ sample collection. If this information is not available, please indicate this.

Not applicable to this study.

How was the potential cultural significance of the materials collected in your study to local communities considered in your research design? Were Indigenous peoples and/or local researchers and institutions involved with archaeological excavations / collection of specimens? If so, please provide a description of their involvement.

Not applicable to this study.

If your manuscript includes photographs of human remains please indicate whether authors obtained permission from descendants or affiliated cultural communities to do so.

Not applicable to this study.
