# Supplementary material for: Urine lipoarabinomannan concentrations among HIV-negative adults with pulmonary or extrapulmonary tuberculosis disease in Vietnam
Source: PLOS Glob Public Health. 2024 Nov 6;4(11):e0003891. doi: 10.1371/journal.pgph.0003891 (PMC11540228; doi:10.1371/journal.pgph.0003891)
Supplement: Attachment — Submitted filename: Response to Reviewers.docx [file pgph.0003891.s009.docx]

**Responses to the Reviewers observations and comments:**

We would like to thank the reviewers for their time and effort to review our manuscript and offering edits and comments that improved the overall quality of our work. We greatly appreciate the opportunity to reply to the reviewer comments raised and it was our intent to clearly address all of them with sufficient detail. Following are our responses to each comment.

**General note:** We have made multiple changes to the text in response to the comments of the reviewers. While the textual changes are tracked, when adding new references, this was done with without using track changes as the document would be very heavily marked and so very hard to read. Where we note the line numbering to address specific points, these are for the document that is in Track Changes. An effect of using line numbering in Rack Changes is that the line numbering can be affected between pages (see pgs 2-3) although while not fully sequential across the draft, each line number is unique.

We added three further references to this manuscript, two (7 and 8) are to address the point raised by Reviewer 1, #2. These are on Line 113. Refs [7-8].

7.	Chatterjee D, Lowell K, Rivoire B, McNeil MR, Brennan PJ. Lipoarabinomannan of Mycobacterium tuberculosis. Capping with mannosyl residues in some strains. J Biol Chem. 1992;267: 6234–6239.

8.	Petzold CJ, Stanton LH, Leary JA. Structural characterization of Lipoarabinomannans from Mycobacterium tuberculosis and Mycobacterium smegmatis by ESI mass spectrometry. J Am

The third reference is to strengthen our Discussion in highlighting an area that may contribute to false positive GeneXpert results. This is on line 681, [33].

33.	Pang Y, Lu J, Su B, Zheng H, Zhao Y. Misdiagnosis of tuberculosis associated with some species of nontuberculous mycobacteria by GeneXpert MTB/RIF assay. Infection. 2017;45: 677–681. doi:10.1007/s15010-017-1044-x.

**Reviewer #1:** Hoa NB et al. studied the use of uLAM levels in HIV-/TB patients as an RDT (using electrochemiluminescent immunoassays), and obtained data are compared to microbiological reference standards. The authors concluded that HIV-negative TB adults (Patients from Vietnam) show low concentrations of uLAM and suggest challenges are imperative for developing a more sensitive rapid uLAM test. The problem approached in this study is relevant worldwide because the search for alternatives for the diagnosis and follow-up of TB patients is necessary to stop TB. Although the topic is very pertinent and the sample size used in the study is very good, this manuscript has several major and minor comments that must be solved before being considered for publication. PLoS Medicine is a journal with high impact, and the authors send a careless manuscript. I suggest that authors consider the below comments, take care of details in the manuscript and submit again.

Major comments

1. The abstract is not clear; several expressions should be defined before use. It should be rewritten.
**Response:** We have revised the abstract with all edits in Track Changes. This should now be clearer.

2. The introduction section should be rewritten because is not clear, for instance:

1) "LAM is a glycolipid in the Mycobacterial cell…" but authors do not clarify if they will search ManLAM (from virulent mycobacteria) or only LAM which could be present in mycobacteria virulent and virulent;

**Response:** Although LAM present in the urine of TB patients is derived from the Mycobacterium, the structure of urinary LAM (referred to as uLAM) is distinct from that isolated directly by the cultured Mycobacteria. Whereas a major fraction of bacterial LAM possesses free dimannose caps (the origin of the term ‘ManLAM’), these are not consistently found in uLAM (see references 20, 38). However, the testing of uLAM from humans has been efficiently captured and quantified with the S4-20 antibody. This is specific for dimannose caps that are terminally substituted with the MTX sugar. We have revised the Introduction section to clarify these distinctions. In addition, to avoid confusion we now refer to LAM from cultured Mycobacteria as cLAM (cultured LAM), and LAM obtained from human participants as uLAM (urinary LAM).

2) TB is caused by M. tuberculosis, and they should explain because if they search LAM, it is also present in M. bovis and it can induce TB under specific conditions. Thus, it is not clear.

**Response:** Various forms of LAM are present from Mycobacterial species, including *M. bovis* and avirulent species. Nonetheless, urinary LAM (uLAM) is a useful marker for TB disease from M. tuberculosis, and was the focus of this study. More specifically, the goal was to quantify LAM in clinical urine samples from HIV-1 negative patients with active TB disease. We have modified the Introduction section to make this clearer.

3. Authors wrote as Exclusion criteria "…having a confirmed TB diagnosis at the time of recruitment" (line 85). So, this means that if the patients have a TB diagnosis was not included even if they do not receive yet an anti-TB therapy?

**Response:** Participants that had a definitive TB diagnosis at time of enrollment were not included in our study. We used this approach in order to retain the cohort design and limit introduction of selection bias during enrollment. This would allow us to make a more accurate inference of the true diagnostic performance in a population of HIV negative adults with presumptive TB.

4. Authors wrote, "…monoclonal capture antibodies.." (line 115). Do they are anti-LAM antibodies? And the same questions to capture antibodies (line 117). I think they are anti-LAM, but authors must indicate it clearly in their methods because readers should not assume.
**Response:** We have revised the text to address this point and make this clear.

5. The Methods section has several abbreviations that are not indicated the mean.

**Response:** We have revised to clarify and define the abbreviations. See line 140 for EPTB, line 181 for MGIT, line 199 for ECL, Line 236 for BSA, move LOD to just after the full name on line 243.

6. Figure 1: What means the "ev" in HIV+ev and HIV-ve? I asked the same question to MRS.
**Response:** Firstly, ‘ev’ was a typo and should have been ‘ve’. We have described the abbreviations to make the text used in Figure 1 and the MRS clearer for the reader. In the document we note participants and HIV-negative and also as HIV-uninfected, we have replaced all HIV-uninfected with HIV-ve once the abbreviation has been noting in the text.

HIV+ve – HIV Positive

HIV-ve – HIV Negative

MRS +ve – TB positive using MRS definition

MRS -ve – TB negative using MRS definition

Each definition is fully described in the manuscript, but abbreviated in Figure 1 for clarity.

7. Do PTBW and EPTBW patients presented essential differences in the blood cell account (mainly immune cells)? This data can be obtained from the data from clinical laboratory tests.
**Response:** Extrapulmonary TB occurs more frequently in immunosuppressed populations compared to pulmonary TB, and studies have demonstrated decreased CD4 levels and PBMC production in patients with confirmed EPTB. However, we are not aware of any studies that have compared immune levels of patients with presumptive TB recruited from either a PTB or EPTB wards. We did not conduct CD4 or CD8 level testing for participants of our study cohort and cannot make inferences concerning the differences in immune function between these two populations.

8. What could be an alternative to improve the sensitivity of the proposed test?

**Response:** We have added some additional potential methods to improve test sensitivity, including urine concentration , enzymatic digestion, and ultrafiltration steps prior to processing, to the Discussion section (lines 749 - 753).

9. Limitations of the study should be indicated.

We have noted the study limitations in the Discussion section (see lines 624 –684).

10. Could this technique be used to quantify LAM in serum? Several tests also are used to evaluate LAM in HIV-, here are discussed several doi: Front Microbiol. 2021 Apr 15;12:638047. doi: 10.3389/fmicb.2021.638047.

**Response:** It may be possible to use these methods to detect LAM in serum. Another group used a similar platform and immunoassays to measure LAM concentration in the serum of patients presenting with TB (PMID: 30998715). However, this was not the focus of our study to investigate the feasibility of accessing urine for use in a rapid triage test for TB in HIV -ve clients.

11. What advantages has this technique compared with other reports to quantify LAM in urine? For instance, Sci Rep. 2023 Jul 18;13(1):11560. doi: 10.1038/s41598-023-38740-3.; PLoS One. 2023 Jul 14;18(7):e0288605. doi: 10.1371/journal.pone.0288605. ACS Nano. 2023 Apr 11;17(7):6998-7006. doi: 10.1021/acsnano.3c01374.; J Clin Lab Anal. 2022 Feb;36(2):e24238. doi: 10.1002/jcla.24238., among others.
**Response:** The primary advantage of using the ECL platform is to quantify uLAM at lower concentrations than most other possible methods, including gas chromatography and ELISA testing. In addition, many prior studies have measured cLAM (LAM from cultured TB), which is different from uLAM (LAM from participant’s urine specimens). This study is not unique for using the ECL assay, but rather for quantifying uLAM concentrations among a large cohort of HIV-negative participants presenting with pulmonary TB-related symptoms in a TB-endemic setting.

In addition, we have added a sentence in lines 171 to 173 , “In the signed consent forms, each participant also agreed to the further use of their urine samples and they are available to other groups developing uLAM assays upon considered request,” in case other groups want to test uLAM from our clinical cohort on their assay platform.

Minor comments

12. What means EPTB (line 35) mean? It is not indicated in the abstract.

**Response:** We have defined this as “extrapulmonary TB (EPTB)” in the text, see line 33.

13. What is S4-20/A194-01 (S/A) (line 36)?

**Response:** This is the antibody combination used to detect TB LAM. We have clarified this on line 35.

14. What is FIND28/A194-01 (F/A) (line 37)?

**Response:** This is a second antibody pair comprised of the FIND28 (capture) and A194-01 (detector) antibody pair (abbreviated as F/A) used in the other immunoassay.

15. The RDT abbreviation used in line 60 has not means, although it was indicated in the abstract, in the manuscript should be indicated the first time that you use it.
**Response:** The RDT abbreviation is defined as “rapid diagnostic test (RDT)” on lines 27 and 118.

16. Tables also need an explanation of used abbreviations.

**Response:** We have added an explanation of the used abbreviations in the tables where pertinent.

17. Language and grammatic need a deep revision.

**Response:** We have reviewed the document and made multiple changes, all visible in track changes.

**Reviewer #2**

This is a well-conducted study on urine lipoarabinomannan concentrations among HIV- uninfected adults with pulmonary or extrapulmonary tuberculosis disease in Vietnam. The study design, datasets, statistical methods and analyses, and presentation (tables and figures) and interpretation of the results are mostly adequate and of a good standard. Only a couple of minor issues needing attention.

1) Table 3 is a key table summarizing the results of test performance against various reference standards. However, could authors please add AUROC with 95% CI for the overall performance for each test?

**Response:** Yes, we have added this to Table 3.

2) Performance comparison is a very important part of the study. The authors have done this in Page 14 and figure 3. However, it would be good to add a Table 4 to summarize these results with all the AUROC, 95% CI and P-values so that it's informative and easy to follow.

**Response:** We have created a Table 4 wherein we summarize sensitivity, specificity and AUC/AUROC comparisons.

**Reviewer #3**

The manuscript "Urine lipoarabinomannan concentrations among HIV-uninfected adults with pulmonary or extrapulmonary TB disease in Vietnam." is an excellent study. The manuscript is well written with appropriate controls and a good sample size. The utility of the assay in detecting u-LAM concentrations is unclear, however, it makes an interesting study, using two different capture antibodies and keeping the detection antibody same, to be used as a diagnostic tool to ascertain the presence or absence of LAM in both pulmonary and extra-pulmonary TB patients, although the number of EPTB sample size is limited. It would be pragmatic to see what effects the urine concentration or any form of pretreatment before the immunoassay is performed would have on the outcome of the study since many published reports have shown that pretreatment helps with the sensitivity of the assay. The authors also highlighted an important point about the geographical origin of samples having an effect on the differences in u-LAM levels. May be a study testing sample cohorts from various TB endemic regions across the world would address that more rigorously. The authors also specified that there were some 35 participants that had NTM co-infection.

Did the co-infection have any effect on the u-LAM levels in those 35 subjects when compared to the TB smear gradation or compared to the remaining cohort u-Lam levels.

**Response:** We have added further text in the Discussion to address this comment.
