# Supplementary material for: Urine lipoarabinomannan concentrations among HIV-negative adults with pulmonary or extrapulmonary tuberculosis disease in Vietnam
Source: PLOS Glob Public Health. 2024 Nov 6;4(11):e0003891. doi: 10.1371/journal.pgph.0003891 (PMC11540228; doi:10.1371/journal.pgph.0003891)
Supplement: Attachment — Submitted filename: PLoS GH Sept Review Editor cover letter.docx [file pgph.0003891.s012.docx]

16^th^ September 2024

Dear Dr Gupta,

We have made the edits that you have recommended in your recent email. These include refining Figure 2 to the specifications that you noted.

“Figure 2 is presented with colours according to MRS status, and separate panels for the different assays and PTB vs EPTB. This would enable the distributions to be compared by MRS status within each panel. There should be 4 panels in total: PTB F/A, EPTB F/A, EPTB S/A, EPTB S/A. We hope this is now clear.”

We also checked this figure and the other images via PACE, all are appropriate. We have also replaced the Abstract with the most current version. We prepared the inclusivity-in-global-research-questionnaire in our original submission, and I have left that in the dossier. As recommended we have also uploaded the latest manuscript in both track changes and in changes accepted.

We would like to thank you for all of your support and patience in this process and we look forward to hearing your response.

Yours sincerely,

David S. Boyle Ph.D

Laboratory Director/Co-lead PATH Diagnostic Program
