# Supplementary material for: Urine lipoarabinomannan concentrations among HIV-negative adults with pulmonary or extrapulmonary tuberculosis disease in Vietnam
Source: PLOS Glob Public Health. 2024 Nov 6;4(11):e0003891. doi: 10.1371/journal.pgph.0003891 (PMC11540228; doi:10.1371/journal.pgph.0003891)
Supplement: Attachment — Submitted filename: Responses to Editorial and Reviewer comments.docx [file pgph.0003891.s010.docx]

**Reviewer comments:**

**In the event that the reviewers do not see our letter of thanks to the Editor, we would like to offer our thanks to the Reviewers too for them taking the time to review and edit our work. We appreciate the considerable effort this takes and so thank you. Your comments have made our work stronger and clearer for the reader.**

**Associate Editorial comments:**

Thank you to the authors for doing a thorough job of addressing the initial reviewers’ critique. The revised article has been reviewed by two peer reviewers. I have provided Editorial comments summarizing the key recommendations for further revisions from both Editorial and Peer Review, along with the full Reviewer comments below. Please consider the summarized Editorial recommendations as essential, and the further full Peer Review comments as for consideration.

**Editorial recommendations:**

I agree with the Reviewer that the “PR-PTBW” and “PR-EPTBW” ward terminology is confusing, particularly because presumptive PTB participants were mostly recruited in an outpatient setting. Suggest rewording throughout as "presumptive PTB" and "presumptive EPTB", without further abbreviation.

**Response:** Revised as recommended.

In the abstract, median uLAM concentrations among the MRS-positives are difficult to interpret with no context. Suggest either removing from abstract, or include comparisons to MRS negative. An indication of how cut-offs were derived would also be helpful in the abstract.

**Response:** We have removed the median uLAM section and added the cut-off details to the abstract.

In the introduction, lines 56-60 are difficult to follow. Suggest rephrasing for a general audience.

**Response:** We have revised this for clarity.

In the methods, some brief details of how demographic and clinical histories were captured would be helpful, e.g. was there a structured participant questionnaire or case record form?

**Response:** We have added further details to better describe this.

Please also clarify whether sputum induction was performed, if spontaneous samples were not possible? And how were trace Ultra results handled?

**Response:** We have added clarification wherein sputum induction was performed if expectorated sputum was unable to be spontaneously produced. Trace positive participants (n=16) were considered Xpert Ultra and MRS positive.

A STARD checklist is strongly recommended.

**Response:** We have added this as a supplementary table; S4 Table.

In the results, please rephrase line 185 as it currently suggests there were “766 participants meetings MRS criteria”.

**Response:** We have to revised the text for clarification.

There is a lot of duplicate information in the Tables. Table 2 could be removed as most of these data are in either Table 3 or Figure 2 (median uLAM concentrations among MRS positives and negatives could be added to Figure 2). I appreciate Table 4 was added in response to a previous Reviewer comment, but since all of this information is now in Table 3, Table 4 could also be removed to avoid duplication and streamline the manuscript.

**Response:** We have removed Table 4. However, we would like to retain Table 2, as it is the only location that provides stratified median uLAM concentration results by stratified by both TB manifestation as well as primary reference classification.

Line 260 suggests that the S/A and F/A data were “pooled”, but it is not clear what this means

**Response:** We have added same text in parentheses to define these.

In line 282, it would be clearer to state “Specificity was higher using F/A…”

**Response:** We have revised the text to state this.

Please avoid overinterpreting point estimates when sample sizes are very small, as highlighted by Reviewer 2 in line 290.

**Response:** We have removed our over interpretation of the data with regard to its small sample size.

**Reviewer 1**

Minor corrections

Inconsistencies:
1. The authors compare the characteristics of enrolled seronegative participants with pulmonary and extrapulmonary presumptions with respect to the values in Table 1; however, the ideas are not supported by the statistical p-values obtained. The authors should show the p-values in the table and indicate the statistical analysis performed in the footnote.

**Response:** Our intent in Table 1 is to describe the demographic and clinical characteristics of participants in the two separate arms, not to draw inferential comparisons between the two groups. Therefore, we do not think that providing p-values is appropriate in this context.

2. The use of "HIV-ve" or "MRS+ev" throughout the manuscript. although in some legends it is observed, I think it would be clearer to leave it in the main text at the level of the introduction.

**Response:** We have now more clearly noted this in the Abstract and now use HIV-negative throughout the manuscript.

3. Previously, it was mentioned that the use of uLAM means the detection of LAM in urine. The text is repetitive, so write a paragraph like “levels of uLAM from urine.”

**Response:** We use the term uLAM is as an abbreviation for the form(s) of LAM that are found in urine, not that LAM was detected in the urinary sample.

Grammatical:
Line 28: Replace “,but there…” with “.However, there is...”

Done.

Line 36: change “,while,” with “. In contrast,”

This sentence was deleted per another reviewers request.

Line 36: Replace “for MRS positive” with “MRS-positive”

This particular text was deleted at the instruction of another reviewer. However, we searched the doc and all MRS positive and negatives were change to the suggested format.

Line 37: Add “the” before “FIND28/A194-01”

This sentence was deleted per another reviewers request.

Line 40: Add “a” before “specificity of 79%”

Done.

Line 49: What is the meaning of “HIV-ve persons”? In the write is not clear where is defined.

We have amended the text to be more clear.

Line 50: Replace “people with TB disease were either not diagnosed” with “TB people were either undiagnosed…”

This sentence was deleted and replaced with different text relating to the mortality rates associated with TB as the lack of Dx and/or referrals is not referenced in the 2023 report.

Line 54: Replace “that has clinical utility for diagnosing TB disease” with “with clinical utility for diagnosing TB.”

Done.

Line 55: Delete “that is also”

Done.

Line 56: Replace “, and while..” with “. While..”

Done.

Line 70: Add comma after “culture” and delete “/or”

We have added a comma but would like to not delete/or as either or both culture and Xpert results were used for the MRS and we feel “and/or” reflects this best.

Line 71: Change “results, but were underpowered..” with “. However, it was underpowered.”

Done.

Line 72: Change “has not been performed..” with “has yet to be performed..”
Done.

Line 77: Change “of” with “in”.

Done.

Line 81: change “adults 18 years of age or older” with “adults > 18 years old”

Done. We used ‘≥’ as participants could be 18 too.

Line 83: Change 6 month with “Six-month”

Done.

Line 88: Change 3 month with “three months”

Done.

Line 89: Add “the” before “time”

Done.

Line 91: Change “be representative of” with “represent people”

Done.

Line 105: Change 6 month with “Six-month”

Done.

Line 108: Replace “For PR-PTBW, we collected separate sputum samples for Xpert MTB/RIF Ultra and Mycobacteria Growth Indicator Tube (MGIT) liquid culture testing for all participants.” With “For PR-PTBW, we collected all participants' separate sputum samples for Xpert MTB/RIF Ultra and Mycobacteria Growth Indicator Tube (MGIT) liquid culture testing.”

Done.

Line 112: Delete “which were”

Done.

Line 112: Change 2 and 6 months with “two and six months”

Done.

Line 114: add “s” at the end of chart.

Done.

Line 115 delete “s” at the end of mL.

Done.

Line 197: a grammatical mistake “PResumed"

Done.

Additional references:

Line 55: Change reference No.4 by <https://doi.org/10.3389/fmicb.2021.638047>
Done, we did not use track changes for this in order to not disrupt all of the references throughout the draft.

Line 56: LAM can be found in serum or plasma. The authors should mention it. <https://doi.org/10.3389/fmicb.2021.638047>
Done, we did not use track changes for this in order to not disrupt all of the references throughout the draft.

**Major corrections**

Inaccuracies:

1. The authors comment that the low sensitivity and specificity of the technique to identify uLAM in HIV-uninfected adults with presumed PTB or EPTB disease is due to factors that are undoubtedly correct. However, the possibility of changes in the structure is not considered in LAM, as the antibodies used do not recognize it. The authors should consider this fact as a point of discussion.

**Response:** We have added further text to the discussion noting this excellent point.

2. It has been reported that storage time can affect the identification and quantification of Lipoarabinomannan (LAM) in urine. This could explain why LAM was not identified in over half of the samples tested under the proposed assay. 10.1183/23120541.00115-2018.

**Response:** This could be a potential reason. However, testing of the same fresh urine samples prior to freezing with an investigational uLAM RDT (although with decreased sensitivity compared to the ECL) yielded similar low diagnostic yield. A further point is that the urine samples were placed at 4°C soon after collection and typically processed and frozen at -80°C within a few hrs with no further thaw until testing via the Mesoscale assay. We also have unpublished data being prepared in a different manuscript regarding repeat testing of a panel of urine samples from FIND that do not show any sign of uLAM decay over time when frozen. We have observed significant decay of uLAM is stored at 4°C for over one 2week but never in the short term.

3. The authors propose that utilizing sample pretreatment methods can aid in releasing LAM, which is often trapped in proteins found in urine. To further support this point, it may be beneficial for the authors to conduct additional experimental trials. An acid hydrolysis procedure, previously reported, could be carried out. <https://doi.org/10.1016/j.aca.2018.09.037>
**Response:** We believe that this study demonstrates that the detectable concentration of uLAM in clinical samples from this specific HIV negative population is very low, and further confirms that uLAM detection in HIV negative populations requires the use of a pre-treatment, pre-concentration step or possibly different antibody combinations that better recognize TB-specific epitopes, and should be explored in future studies. We did assess using proteinase K treatment on urine samples in earlier work. However, we did not see any significant improvement in the detection of uLAM by this method.

Unfortunately our funding ended over a year ago and we do not have any further resources to look at further pretreatment methods although we agree with the reviewer, they will be undoubtedly necessary for samples like these and we note some of these in the discussion. It was one of the reasons why we were keen to freeze large aliquots to help developers assess their tools that purify or treat the urine samples in order to improve assay sensitivity.

**Additional comments:**

In lines 75-77, the authors should indicate the total number of this study cohort, showing the total numbers of PTM and EPTB patients.

**Response:** This is now highlighted in the results section.

In the section titled “Evaluation of clinical urine specimens using immunoassays,” the authors should mention the epitope that both antibodies FIND28 and S4-20 recognize in LAM. Also in this section, the authors mentioned that the LAM used for the standard curve was derived from the in vitro culture of the TB strain Aoyama B. The authors should explain or shows evidence to select this strain considering changes in LAM structure and specificity of antibodies compared with other strains.
**Response:** We have added some text and a reference to describe the epitopes that this pair and A194-01 recognizes the Aoyama B strain is lineage 2. We chose it because the original assay by Sigal et al. was already described in the literature as being the most sensitive uLAM assay currently available and therefore we did want to change the standard which would render the previous data sets incomparable. In a previous publication (referenced in this work), we did assess the effects of cultured LAM from different lineages of Mtb on a wide range of candidate antibodies (PMCID: PMC9524686). In that study we saw no significant differences with the performance of multiple antibodies screened on cultured LAM purified from lineages 1-4 including the pairs of F/A and S/A.

In lines 50-51, the statistical of people with TB disease should be updated, considering the latest WHO report (2023).

**Response:** The 2023 report does not note the discrepancies seen with undiagnosed and/or under reporting of diagnosed cases of TB infection. We have changed the language to instead reflect the overall global burden of TB disease and the mortality rates observed with PLHIV who are adults.

**Figures and Tables:**

Figures 2 and supplementary figure 1: Information associated with the colors in the graph needs to be included. There is no legend describing the image observed.

**Response:** We have added text to the figurehead for Figure 2. We now explain what the contents of the table are in this. The figure head of supplemental figure 1 is placed separately in the document. It can be found with the others on page 28

Reviewer #2: This is a well written and methodologically rigorous paper which addresses an important clinical question and reports useful negative findings. I suggest a few minor revisions to improve the clarity and readability of this paper.

Firstly, the use of terminology throughout the paper needs to be addressed. Terminology on disease states should be brought in line with the TB-ICE consensus classification (<https://doi.org/10.1016/S2213-2600(24)00028-6>) and the reference to wards in PR-PTBW and PR-EPTBW is unclear and should be clarified.
Secondly, it is unclear what threshold/cut-off has been used to calculate performance metrics throughout and why this cut-off was used. AUROC is a better performance metric as takes into account different thresholds. The optimal/minimal WHO Target Product Profiles are not mentioned in the manuscript but must be as a benchmark. Finally the link to the publicly accessible data on Dataverse is not working.

**Responses:** Terminology has been updated to reflect TB-ICE classification, and PR-PTBW and PR-EPTBW have been changed to presumptive TPB and presumptive EPTB respectively. We have revised the text to describe the methodology used; we applied the threshold determination as described by MSD. Reference to the WHO TPPs for priority TB diagnostic tests including one for non-sputum-based biomarkers has been added to the discussion. The link to Dataverse is now active.

Below are some further detailed suggestions:
Line 29 - Switch from "TB-related symptoms" to suspected clinical TB throughout

**Response:** We disagree with the use of “suspected clinical TB”, as this does not reflect the use of non-stigmatizing, patient-centric language. We have revised the wording to “clinical TB-related symptoms” for clarification without use of “suspected”.

Line 41 - Make clear that these are AUROC values
**Response:** We have done this.

Line 64 - Sentence starting 'As such, the only...' does not follow from the previous sentence and should be amended/deleted

**Response:** This is in reference to the RDT (Alere Determine TB LAM Ag) described in the previous sentence.

Line 71 - Suggest elaborating on the mixed results in reference 21

**Response:** We have revised this.

Line 86 - The TB ward terminology is confusing, especially considering some were recruited from outpatients. Suggest switching throughout to suspected PTB and suspected EPTB.

**Response:** We have revised as suggested.

Line 164 - For the CRS did you address patients lost-to-follow-up? Were there any patients LTFU? If not, should be clarified in the results.

**Response:** For this specific analysis, TB treatment by month 2 was considered positive by the CRS. LTFU was low by the 2M visit. Of the 9 participants with a missing 2M FU form, 4 participants were MRS positive at baseline, 1 participant identified as having non-tuberculosis mycobacterium and 2 were empirically initiated on treatment at baseline. 1 participant was excluded from analysis due to their urine sample being collected outside of the collection time window. 1 participant was later reached at their 6M visit, but indicated they were still TB negative and had not initiated TB treatment. For the final participant not reached at 2M, records indicated that they had died 5 months after their initial baseline visit, and clinical chart review and laboratory review of records did not identify interim TB treatment.

Line 171 - Switch from "presented for additional information" to "secondary analyses".

**Response:** Switched.

Line 176 - Unclear when unpaired vs paired ROC comparisons were performed. Paired comparison should be used.

**Response:** Revised for clarity. We used unpaired ROC comparisons when comparing individual antibody performance between the presumed PTB & presumed EPTB groups, as these were independently recruited groups requiring slightly different sampling for testing due to their disease presentation.

Line 180 - The link to the publicly accessible code is not working, hence unable to verify if all data is available

**Response:** The link to Dataverse remains nonfunctional until the manuscript is published by PLoS.

Line 207 - How did you address those with both pulmonary and extra-pulmonary TB - i.e. the 7.5% of EPTB pts with a positive CXR? How were they classified?

**Response:** Participants recruited to one group were not reclassified as having confirmed PTB or EPTB unless a positive Xpert Ultra or MGIT culture result was obtained using a respiratory or extrapulmonary specimen, respectively**.** This was the case for only 1 participant recruited to the presumptive EPTB group that was reclassified to the PTB group. Due to the difficulty in testing extrapulmonary specimens using Xpert Ultra cartridges, there were several participants that had a respiratory specimen taken for Xpert testing & extrapulmonary specimen taken for culture testing, with their testing on respiratory specimens being negative. However, the limitations of this approach are acknowledged in the discussion (L447)

Line 293 - The abbreviations in this sentence are not clear.

**Response:** In looking at ln 293 I only see S/A which is routinely used in the text after the antibody pair was first described.

Line 223 - In table 1, switch from "History of recent TB exposure" to "Household contact". Additionally "Culture positive" and "CRS" should be separate rows.

**Response:** We have made this change.

Line 224 - It is unclear what being compared in this sentence.

**Response:** We have re written the sentence to note the difference between the F/A Ab pair and the S./A Ab pair.

Line 253 - It is not clear what threshold/cut-off has been used to calculate the accuracy throughout the results. This should be clarified with reasoning as to why it was chosen.

**Response:** We have revised the text for greater clarity.

Line 260 - Sentence not clear, review.

**Response:** This sentence has been amended and the referral to Table S2 and replaced with Table S1.

Line 274 - Table 3 is very large and may be better in the supplementary materials, with a smaller table containing salient results (N, sensitivity, specificity, AUROC) in the main manuscript. Alternatively the entire table can be moved to supplementary and Table 4 expanded to include CRS.

**Response:** While we agree with the reviewer on large the size of this table, we would like to keep it in its current format as it contains all of the performance data that underpins our work. Placing some of the data in a supplemental table can means the data there is often ignored as the reader has to further access the S tables.

Line 286 - Some subheadings would be useful in the results section.

**Response:** Added for clarity

Line 290 - I do not think "appeared to be slightly higher" is appropriate with n=6. Suggest rephrasing this sentence to something like "a comparison of Ab pairs was limited in the EPTB MRS positive group which contained only 6 individuals (50% vs 33%, p>0.99)".

**Response:** We have removed this result given the very low number of participants with that result.

Line 296 - In Figure 3 I suggest that this is split into two side-by-side plots for PTB and EPTB, and 95% CI are added.

**Response:** Figure 3 has been revised to reflect suggestion.

Line 317 - Pivot from "presumptive TB disease" to "presumed clinical TB" in line with ICE-TB consensus classification (<https://doi.org/10.1016/S2213-2600(24)00028-6>).

**Response**: Revised as suggested.

Line 319- The AUROC is more illustrative of the performance of the test across different thresholds and should be mentioned before sensitivity/specificity here.

**Response:** Description of results is ordered to match order in which presented in Table 3, and we believe it makes sense to first highlight the sensitivities and specificities first to illustrate the readers.

Line 322 - Subclinical-TB is a reference to absence of signs/symptoms as per ICE-TB consensus classification so suggest changing to paucibacillary disease.

**Response:** Revised as suggested.

Line 421 - This is the first time the Target Product Profiles are mentioned. This needs to be included in the introduction, with abbreviation defined.

**Response:** Revised
