# Supplementary material for: Urine lipoarabinomannan concentrations among HIV-negative adults with pulmonary or extrapulmonary tuberculosis disease in Vietnam
Source: PLOS Glob Public Health. 2024 Nov 6;4(11):e0003891. doi: 10.1371/journal.pgph.0003891 (PMC11540228; doi:10.1371/journal.pgph.0003891)
Supplement: Attachment — Submitted filename: 8 14-24 Responses to Editorial and Reviewer comments.docx [file pgph.0003891.s011.docx]

8/14/2024

**Responses to reviewers.**

Dear Editor and Reviewers,

Please find our repsonses to your comments and suggested edits below. We thank oy for your time and have made the recommended changes to each.

Best regards,

David

1. As highlighted by the Reviewer, there is some residual use of the "wards" terminology and abbreviations, including in the Abstract.

**Response:** We have made these changes in Track Changes and they can be followed throughout the document

2. The new sample size statement (line 196) is ambiguous and requires clarification - what diagnostic accuracy metrics is this based on?

**Response:** We revised the text around ln196 to better describe this. The specific text is

“Receiver operating characteristic (ROC) curves were calculated to evaluate the overall discriminatory ability of each antibody (Ab) pair, and statistically compared using paired ROC comparisons(R package pROC, v1.18) when comparing performance between the same participants. [25]. We used unpaired ROC comparisons when comparing between presumptive PTB & presumptive EPTB individuals.”

3. In Figure 2 (the density plots), these may benefit from being stratified by the primary MRS status.

**Response:** We have made this change and have uploaded a revised figure that reflects this.
